# Planned and unplanned hospital admissions and health-related school absence rates in children with neurodisability: Protocol for a population-based study using linked education and hospital data from England.

**DOI:** 10.3310/nihropenres.13558.1

**Published:** 2024-05-01

**Authors:** Laura Gimeno, Ania Zylbersztejn, Ayana Cant, Katie Harron, Ruth Gilbert

**Affiliations:** 1 Great Ormond Street Institute of Child Health, University College London, London, England, WC1N 1EH, UK; 2 Centre for Longitudinal Studies, Social Research Institute, University College London, London, England, WC1H 0NU, UK; 3 NIHR Great Ormond Street Hospital Biomedical Research Centre, Great Ormond Street Hospital Children's Charity, London, England, WC1N 3JH, UK

**Keywords:** neurodisability, school absence, hospital admissions, electronic health records, linked data

## Abstract

**Background:**

Neurodisability describes a broad set of conditions affecting the brain and nervous system which result in functional limitations. Children with neurodisability have more hospital admissions than their peers without neurodisability and higher rates of school absence. However, longitudinal evidence comparing rates of hospital admission and school absence in children with neurodisability to peers without neurodisability throughout school is limited, as is understanding about whether differences are greatest for planned care (e.g., scheduled appointments) or unplanned care. This study will describe rates of planned and unplanned hospital admissions and school absence due to illness and medical reasons throughout primary school (Reception to Year 6, ages 4 to 11 in England) for children with neurodisability and all other children, using linked individual-level health and education data.

**Methods:**

We will use the ECHILD (Education and Child Insights from Linked Data) database, which links educational and health records across England. We will define a primary school cohort of children who were born in National Health Service-funded hospitals in England between 1 ^st^ September 2003 and 31 ^st^ August 2008, and who were enrolled in Reception (age 4/5) at state-funded schools. We will use hospital admissions records to identify children who have recorded indicators of neurodisability from birth up to the end of primary school (Year 6, age 10/11).

**Results:**

We will describe rates of planned and unplanned hospital admissions and health-related school absence for three groups of children: those with a neurodisability indicator first recorded before beginning primary school, those with neurodisability first recorded during primary school, and those without a record of neurodisability before end of primary school.

**Conclusions:**

We will further explore whether differences between these group vary across primary school years and by socioeconomic and demographic characteristics.

## Plain English summary

Neurodisability encompasses a range of health conditions which affect the brain and nervous system and result in difficulties with everyday activities, including learning. Children with neurodisability are more likely to be admitted to hospital and spend longer periods of time in hospital than children without neurodisability. They are also more likely to be absent from school. Yet, in England, these is a lack of evidence comparing admissions and absence rates in children with and without neurodisability throughout their school years. Evidence is also lacking on whether differences are greatest for planned care (e.g., scheduled appointments) or unplanned care. We will use hospital and education records from state-funded hospitals and schools in England to describe rates of hospital admission and school absences for children with and without neurodisability during their primary school years.

## Introduction

Neurodisability encompasses a range of “congenital or acquired long-term conditions that are attributed to impairment of the brain and/or neuromuscular system and create functional limitations” ^1^. Neurodisability includes neurodevelopmental disorders such as learning disability, autism spectrum disorder (ASD) and attention-deficit/hyperactivity disorder (ADHD), neurological conditions such as epilepsy and cerebral palsy, and a broad set of other conditions affecting learning and brain development (e.g., chromosomal anomalies, paediatric stroke, and brain tumours).

Many of the individual conditions encompassed under this definition of neurodisability are rare. For instance, 2–3 livebirths per 1000 are affected by cerebral palsy ^2^, and 1.2 livebirths per 1000 by Down syndrome ^3^. This relatively small group of children have a much greater need for healthcare compared to their peers. For example, in Northern Ireland, children with cerebral palsy make up only 0.3% of the population aged 0–24 years, but account for 1.6% of all hospital admissions and outpatient appointments in this age-group ^4^. A number of record linkage studies, both within the UK (England, Wales, Scotland) and internationally (Australia) have shown that children with a variety of neurodisability subtypes, including neurological conditions such as cerebral palsy and epilepsy ^4– 8^, Down syndrome ^9^, and neurodevelopmental conditions, such as ADHD ^10, 11^, ASD, and learning disabilities ^12^, are more frequently admitted to hospital and for a longer duration than their peers without neurodisability.

Children with neurodisability are also more likely to be absent from school ^8, 11, 13, 14^. There is some evidence among children with learning difficulties and ASD that these higher rates of absence may be driven by their greater need for healthcare ^15^, resulting in more time away from school. However, this evidence is cross-sectional, based on published aggregate statistics, and relies on children being in receipt of Special Educational Needs support to be identified as having learning difficulties or ASD, which not all children with neurodisability receive. Longitudinal evidence on how health-related school absence rates change over the course of primary school for children with neurodisability more generally is limited. While evidence for the role of school absence as a mediator for associations between chronic health conditions like neurodisability and school attainment is weak ^16^, education remains a key social determinant of many health and socioeconomic outcomes in adulthood. It is therefore important to understand how the complex healthcare needs of children with neurodisability affect schooling.

In this study, we will use linked education and hospital records to quantify rates of planned and unplanned hospital admissions and health-related school absence during primary school (age 4/5 to 10/11 years) in England, for children with and without neurodisability. Little research has explored whether differences in admissions and absences between children with neurodisability and their peers are greatest for planned care, reflecting proactive management of the complex medical conditions of children with neurodisability, or unplanned care. We will also explore whether differences in admission and absence rates between children with neurodisability and their peers differ by school year and by socioeconomic and demographic characteristics (area-level deprivation, recorded eligibility for free school meals, ethnicity, geographic region, and month of birth).

This study is part of the wider Health Outcomes of young People in Education (HOPE) research programme, which aims to understand the impact of Special Educational Needs (SEN) provision of children and young people’s health and education outcomes. The umbrella protocol for the HOPE research programme has been published elsewhere ^17^.

## Methods

### Ethics and dissemination

Permissions to use linked, de-identified data from Hospital Episode Statistics and the National Pupil Database were granted by the Department for Education (DR200604.02B) and NHS Digital (DARS-NIC-381972). Ethical approval for the ECHILD project was granted by the National Research Ethics Service (17/LO/1494), NHS Health Research Authority Research Ethics Committee (20/EE/0180), and UCL Great Ormond Street Institute of Child Health’s Joint Research and Development Office (20PE06). Access to the ECHILD database is approved by the ECHILD team ( ich.echild@ucl.ac.uk) for proposals and projects using ECHILD.

Findings will be disseminated to stakeholders (including academics, government departments, service users, and service providers) through seminars, workshops, and peer-reviewed publications. We will publish the code used for analysis in an open-source repository to enable others to replicate and build upon our work using ECHILD.

### Study type

This is an observational study, using linked health and education records to conduct a population-based birth cohort study.

### Dataset and linkage

The ECHILD (Education and Child Health Insights from Linked Data) database contains linked administrative data on health and education for approximately 14.7 million children and young people born in England between 1 ^st^ September 1995 and 31 ^st^ August 2020 from age 0 to 24 ^18^.

Health data comes from Hospital Episode Statistics (HES) for England, a database which records contacts with all National Health Service (NHS) funded hospitals. In this study we will use HES Admitted Patient Care (APC) datasets, which record all inpatient episodes in NHS-funded hospitals since 1997 ^19^. HES records contain basic demographic information and information of diagnoses (coded using International Classification of Diseases 10 ^th^ Revision (ICD-10) codes) and procedures (coded using Office of Population Censuses and Surveys Classification of Interventions and Procedures (OPCS-4) codes). HES APC contains information on birth admissions which can be used to construct birth cohorts from administrative data. NHS England produces study-specific pseudonymised patient identifiers which can be used to link hospital admissions in the same individual over time, through further admissions and access to other health services. Coverage of HES is high, since most secondary care in England occurs in NHS or NHS-funded hospitals (98–99%), and nearly all children born in England (97%) have a birth record in HES ^19^. In the ECHILD database, HES data is linked to Office for National Statistics (ONS) Mortality Data for deaths from 1 ^st^ January 1998 onwards, enabling us to capture deaths that occur outside of the hospital (in hospital deaths are captured in HES).

Education data comes from the National Pupil Database (NPD), which contains information of registration, attainment, absences, and exclusions of children attending state-funded schools in England ^20^. The Department for Education (DfE) produces study-specific anonymised Pupil Matching Reference (aPMR) numbers which can be used to link education records for the same individual across their school careers. During primary school years (Reception to Year 6 in England), NPD only captures children who are registered at state-funded schools. It is estimated that 7% of children in school each year in England attend independently funded (private) schools ^20^, and that 0.5-1% are home-schooled ^21, 22^.

HES and NPD records are deterministically linked by NHS England using an algorithm which uses identifiable information (including name, date of birth, sex, and postcode) to create a bridge file allowing researchers to link pseudonymised patient identifiers and aPMRs ^18^, enabling the creation of longitudinal educational and healthcare histories for each individual in our study cohort. The linkage rate between HES and NPD is high and has improved with time (94–98%) ^18^.

### Study population

The study population consists of all singleton children born in NHS-funded hospitals in England between 1 ^st^ September 2003 and 31 ^st^ August 2008 (academic years 2003/4 to 2007/8) who were linked to NPD and recorded as enrolled in Reception at state-funded schools (age 4/5) in the January (Spring) School Census. We focus on children born during this period since these children would be expected to have completed primary school (end of Year 6, age 10/11) by 31 ^st^ August 2019 ( Table 1). This was the last academic year that was unaffected by the COVID-19 pandemic, which began in March 2020. Lockdowns during the COVID-19 pandemic affected children’s access to school, and the frequency of planned admissions and outpatient appointments reduced substantially during the pandemic ^23, 24^. We use the January School Census since it is used for the allocation of school funding (and so is assumed to be the most complete). While mandated primary school begins in Year 1 (age 5/6) in England, most children are also enrolled in Reception, beginning school at age 4/5.

**Table 1.** Timeline for primary school cohort.

		Academic Calendar Year															
		2003/4	2004/5	2005/6	2006/7	2007/8	2008/9	2009/10	2010/11	2011/12	2012/13	2013/14	2014/15	2015/16	2016/17	2017/18	2018/19
**Academic Year of Birth**	2003/4	**0**	1	2	3	4	5	6	7	8	9	10	**11**	12	13	14	15
	2004/5		**0**	1	2	3	4	5	6	7	8	9	10	**11**	12	13	14
	2005/6			**0**	1	2	3	4	5	6	7	8	9	10	**11**	12	13
	2006/7				**0**	1	2	3	4	5	6	7	8	9	10	**11**	12
	2007/8					**0**	1	2	3	4	5	6	7	8	9	10	**11**

**Legend:**
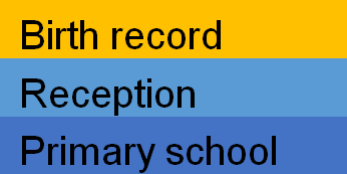
*Note:* The number in each box indicates the how old a child would turn on their birthday in given academic year. Mandated primary school years are Year 1 to Year 6, although many children enter in the year they turn five (referred to as Reception year). Academic years are from 1 ^st^ September to 31 ^st^ August the following year.

Children will be excluded from the study cohort if their NHS record does not link to NPD (likely indicating they did not attend state-funded school in England, that they died or emigrated before primary school age, or a missed link) ^25^, or if they did not appear in Reception (age 4/5) in any January School Census. We will also exclude children who are registered two or more years outside of their expected school year, based on their year of birth.

### Follow-up

Children will be followed from Reception (age 4/5) until the end of primary school (Year 6, age 10/11), death, or the end of the study (31 ^st^ August 2019), whichever occurred first. Follow-up time will be split by school year (from 1 ^st^ September to 31 ^st^ August).

For hospital admissions, we will follow children across the whole of their primary school years, from Reception until end of Year 6 or death. Children’s hospitalisation rates can be characterised across their primary school years whether they are in state-funded school (and so feature in a school census in NPD) or not. Loss to follow-up from HES can occur because of emigration, but due to a lack of data on migration, a limitation of this study is that we will not be able to censor emigrants.

For absences, outcome data is only available for those enrolled in a state-funded school in a given academic year. A child initially enrolled in state-funded Reception, and so part of the primary school cohort, may not appear in NPD in subsequent years for a variety of reasons including emigration, transition to home-schooling or independent non-state schooling, off-rolling, or death before the end of primary school. We will split follow-up time by school year, and for each school year consider only children who are enrolled. If a child subsequently reappears in a school census having been previously missing (provided a death has not been recorded), then they will be reincorporated into the analytical sample for that school year. We will provide information on the percentage of children in the primary school cohort who are recorded in NPD for each school year, and so factor into absence rate calculations for that year.

### Exposure: neurodisability

We will use children’s hospital admission and mortality records from birth up to the 31 ^st^ August of Year 6 (age 10/11) to identify children with neurodisability based on ICD-10 diagnostic codes and OPCS-4 procedural codes. The codes used to identify cases were collated from published papers and code lists and compiled in collaboration with clinicians. The methods used to identify children with neurodisability in HES according to these code lists will be published elsewhere ^26^, and the code lists themselves will be made available in an online repository. Using these codes, we will create three exposure groups: children who had an indicator of neurodisability first recorded before the start of primary school (i.e., before 1 ^st^ September of Reception), children who had a first record of neurodisability during primary school (i.e., between 1 ^st^ September of Reception and 31 ^st^ August of Year 6), and those who had no recorded codes indicating neurodisability before the end of primary school.

The definition of neurodisability in our study follows the consensus definition proposed by Morris and colleagues: “Neurodisability describes a group of congenital or acquired long-term conditions that are attributed to impairment of the brain and/or neuromuscular system and create functional limitations. A specific diagnosis may not be identified. Conditions may vary over time, occur alone or in combination, and include a broad range of severity and complexity. The impact may include difficulties with movement, cognition, hearing and vision, communication, emotion, and behaviour” ^1^. Following this definition, conditions identified as a neurodisability include neurodevelopmental disorders (e.g., learning difficulties, ASD, ADHD), neurological disorders (epilepsy, cerebral palsy), genetic conditions likely to affect learning (e.g., Down syndrome, sex chromosome anomalies), musculoskeletal disorders (e.g., spina bifida and anomalies of the spinal cord), and conditions which affect the brain (e.g., paediatric stroke, hydrocephalus, inflammation of the brain, brain tumours), and perinatal conditions affecting the brain (e.g., neonatal abstinence syndrome/foetal alcohol syndrome, perinatal brain injury). Our definition excludes traumatic brain injuries and other acquired injuries to the head since head injury is common, but severity and resulting functional limitations are not well captured in hospital records.

### Outcome: planned and unplanned hospital admission

Our analysis will focus on planned and unplanned hospital admissions to state-funded hospitals in England. Our outcome therefore reflects more severe healthcare contacts: planned interventions requiring admission to hospital (rather than planned care received in the community, primary care, or at hospital outpatients), and unplanned health events resulting in hospital admission (rather than any contact with emergency departments). 

We will extract data on all hospital admissions for all children in the cohort during their primary school years: from 1 ^st^ September of Reception (age 4/5) to 31 ^st^ August of Year 6 (age 10/11), or death. Admissions are continuous periods in hospital that could consist of several finished consultant episodes (a period of hospital stay under a single consultant). Admissions within one day of each other (discharged and re-admitted on the same or following day) or admissions that included a hospital transfer will be considered as a single admission. We will classify admissions into planned (elective) and unplanned (emergency) admissions using the admission method of the first episode within the admission.

### Outcome: school absence

Data on number of absent sessions are collected every term throughout primary school. For the purposes of this study, we will not differentiate between authorised and unauthorised absences. Instead, we will count total absences, measured as a percentage of available school half days that the pupil was absent, and persistent absence (absent from ≥10% of possible sessions during the school year) ^28^. We will also analyse the subgroup of health-related absences, recorded as “due to a doctor or dentist’s appointment” (henceforth referred to as medical absences), or “due to illness”.

### Additional variables

Results will be presented stratified by sex at birth recorded in HES and school year (the academic year runs from 1 ^st^ September to 31 ^st^ August the following year). School year will be determined based on the school year indicator recorded in each January School Census: Reception (age 4/5) to Year 6 (age 10/11). We will assume that the small number of children with missing data on school year are in the expected school year for their date of birth.

In secondary analyses, we will explore whether differences in hospital admission and absence rates between children with neurodisability and their peers vary by five socioeconomic and demographic characteristics, measured at school entry (January School Census of Reception, age 4/5). If data are missing in Reception, we will use the earliest complete recoding available in any subsequent January School Census (usually Year 1), under the assumption that these characteristics are unlikely to change during primary school. Considered sociodemographic indicators are:

1. Income Deprivation Affecting Children Index (IDACI) quintile associated with the pupil’s residential address. IDACI is an area-level measure of the proportion of children under the age of 16 living in low-income households. We will retain a separate category for children with missing IDACI.
2. Whether a pupil was recorded as eligible for free school meals (yes/no).
3. Government Office Region of residence associated with the pupil’s residential address (North East, North West, Yorkshire and the Humber, East Midlands, West Midlands, East of England, London, South East, South West, or missing).
4. Mode of ethnicity across School Censuses (Asian or Chinese, Black, Mixed, White, any other ethnic group, or unclassified).
5. Child’s birth month, using birth date recorded in HES birth admissions.

### Statistical analysis

***Descriptive analysis.*** We will describe the characteristics of the primary school cohort in Reception (age 4/5) for each exposure group (i.e., children with neurodisability first recorded before primary school, those with neurodisability first recorded during primary school, and those with no recorded neurodisability from birth to the end of primary school). We will give further information on the distribution of children with neurodisability in the cohort by neurodisability subtype recorded in health records (e.g., ASD, cerebral palsy, perinatal conditions affecting the brain, chromosomal anomalies).

For children with and without neurodisability, we will describe the proportion of children in the primary school cohort who appear in NPD for each school year, and the percentage of children who died before the end of primary school (31 ^st^ August of Year 6, age 10/11, ascertained through linkage to ONS mortality data or discharge method in HES).

***Planned and unplanned admissions*.** Results for children with neurodisability recorded before start of primary, during primary school and with no record of neurodisability before end of primary school will be presented by school year (Reception to Year 6), overall and stratified by sex.

We will calculate rates of planned and unplanned hospital admissions by dividing the total number of planned and unplanned admissions during a given school year by total person-time at risk (expressed in days) in the academic year. For each cohort member, time at risk during a given academic year is the number of days between 1 ^st^ September and the earliest of either 31 ^st^ August the following year or death, minus time spent in admitted patient care (APC). We discount time spent in APC since a child cannot be at risk of readmission to hospital if they are already admitted.

We will calculate the proportion of children with ≥1 planned and ≥1 unplanned hospital admission, by sex and school year, by dividing the number of children with at least one admission during that school year by the total number of children alive at the start of the school year.

Finally, we will calculate the proportion of all days that children spend in hospital during primary school (for planned and unplanned admissions) which are contributed by children with neurodisability.

In addition to visualising these outcomes, we will use regression models to quantify relative differences in planned and unplanned hospital admission between children with neurodisability (recorded before primary school and during primary school) and their peers without recorded neurodisability, adjusting for socioeconomic and demographic characteristics and school year. In secondary analyses, we will explore whether differences between these groups widen or narrow over the course of primary school by testing for interaction between neurodisability and school year. Finally, we will describe whether differences between children with and without neurodisability vary by socioeconomic and demographic characteristics, by testing for interactions between indicators for these characteristics and neurodisability.

***Absences*.** Results for children with neurodisability recorded before start of primary school, during primary school and with no record of neurodisability before end of primary school will be presented by school year (Reception to Year 6), overall and stratified by sex.

We will calculate rates of school absence (overall, medical, due to illness, and health-related) by dividing the number of absences in the school year by the total possible number of sessions among enrolled children during that school year.

We will calculate the proportion of children with persistent absence (≥10% of absent sessions) by dividing the number of children with a flag for persistent absence by the number of children registered in that school year in the January School Census.

As well as visualising these outcomes, we will use regression models to quantify relative differences in absence rates between children with neurodisability (recorded before primary school and during primary school) and their peers without neurodisability, adjusting for socioeconomic and demographic characteristics and school year. As described for hospital admissions, we will also explore whether differences in absence rates between these groups widen or narrow over the course of primary school, and whether they vary by socioeconomic and demographic characteristics.

### Bias

In some cases, children with neurodisability may be misclassified, since we assume that if a child does not have a code indicative of neurodisability in their hospital records before the end of Year 6 (age 10/11), they do not have neurodisability and are included in the ‘all other children’ comparator group. We also note that children whose codes indicating neurodisability that are first recorded in HES during primary school may have been diagnosed before primary school.

In both instances, children with neurodisability who are misclassified are more likely to be those with milder forms of neurodisability who are likely to receive diagnosis and healthcare in primary care and community paediatrics settings. Since we expect that children with neurodisability (even in its milder forms) are likely to have worse outcomes than their peers without neurodisability, we expect that our analysis may underestimate the difference between children with neurodisability and all other children. Rates of hospital admission and school absence themselves may be overestimated, since children included in the neurodisability group are likely to have more severe versions of neurodisability than those who were not identified.

### Sensitivity analyses

We will provide additional information on the characteristics of children in the primary school cohort compared to all children born in English NHS-funded hospitals between 1 ^st^ September 2003 and 31 ^st^ August 2008. We will also explore whether results differ when further stratifying by academic year of birth, to check whether improvements in coding and diagnosis of disability across cohorts have substantially affected our findings.

### Strengths, limitations, and opportunities for further research

Strengths of this study will include its use of linked health and education data covering a large number of children born in England across several years (forming several school year cohorts). Linkage of health and education data means that children with neurodisability can be identified by their hospital records. The large sample size means that differences in admissions and absence rates between children with and without neurodisability recorded before end of primary school can be stratified by socioeconomic and demographic indicators.

This study will also has limitations. Our analysis will focus on hospital admissions, rather than healthcare contacts more widely. Not all healthcare contacts resulting in health-related absences are recorded in HES. While outpatient data has been recorded since 2003/2004 and is available in HES, many other types of planned care are not included. Contacts with primary care and community paediatrics will not be recorded in HES, and children with neurodisablity attending special schools may receive additional care at school, which will also be missed. Accident and Emergency (A&E) data in HES was experimental until 2012/2013, and the percentage of attendances captured remained <85% until 2014/15 ^27^, such that no cohort included in our study had A&E data available from Reception. Since our analysis focuses on hospital admissions, reflecting more serious planned interventions or unplanned health problems which are not easily dealt with in primary care, rather than healthcare contacts more generally, interpretation of our results is less impacted by certain types of care not being recorded in HES. However, since this study treats health-related school absence as an outcome, the impact of healthcare contacts beyond hospital admissions on education will still be captured to some extent.

Finally, our research will describe differences between children with neurodisability and their peers for hospital admissions and absences separately. Further research may seek to explore the extent to which health-related absences in NPD can be explained through hospital contacts captured in HES.

## Data Availability

No data are associated with this article.
