# Supplementary material for: Planned and unplanned hospital admissions and health-related school absence rates in children with neurodisability: Protocol for a population-based study using linked education and hospital data from England
Source: NIHR Open Res. 2024 May 1;4:26. [Version 1] doi: 10.3310/nihropenres.13558.1 (PMC11319901; doi:10.3310/nihropenres.13558.1)
Supplement: Peer review history

# Reviewer report: Reviewer response for version 1

**DOI:** 10.3310/nihropenres.14717.r32442

**Published:** 2024-08-14

**Authors:** Simon Paget

**Affiliations:** 1 The University of Sydney, Sydney, New South Wales, Australia

Thank you for the opportunity to review this manuscript. The authors describe a protocol for a data linkage study to examining relationships between (planned and unplanned) hospital admissions and school absences in primary school-aged children with and without neurodisabilities.

 The protocol is well written, and generally clear and easy to follow.

 The methodology to ascertain the population (i.e., children born in an NHS hospital between 1/9/2003 and 31/8/2008, and enrolled at (state-funded) school at age 4/5) using administrative data appears robust, and the small number of children missed in this way (e.g., through migration, private school) are unlikely to negatively impact the study’s findings.

 Identifying children with neurodisability from this population will be more challenging using the proposed methodology i.e., ICD-10 codes from hospital admission data. It seems likely that many of the children in the (total population) with more common neurodisabilities (e.g., learning disabilities, attention deficit hyperactivity disorder (ADHD), autism spectrum disorders) may have either never been admitted to hospital during the study period (e.g. ADHD), or have been admitted for another reason, in which case the ICD-10 codes may not be accurate (i.e., diagnoses missed) (e.g. a child with ADHD admitted with appendicitis). As the authors note, this methodology is likely to skew to a more severe neurodisability population (e.g. see a recent article on CP = Paget SP, McIntyre S, Lain S, Goldsmith S, Nassar N. A comparison of cohorts of children with cerebral palsy from a population register and hospital admission data: A data linkage study. Paediatr Perinat Epidemiol. 2024; 38: 22-30. doi:10.1111/ppe.13024), and perhaps not ascertain comorbidities well. Of note here, the most common causes of hospitalisation here often are unrelated to the neurodisability but are due to common comorbidities (e.g., greater risk of respiratory illnesses, epilepsy). I note the plan to index the code lists, which is welcome.

 I think that one issue that the authors should consider more is to explicitly state the specific research question this study aims to answer. This doesn’t feel clearly articulated in the protocol at present. For example, the abstract and introduction provide evidence that children with neurodisability are already known to have a higher frequency of hospital admissions and school absences than the general population, so the rationale to repeat that in this study is perhaps unclear. The Methods section suggests two outcomes i.e., hospital admissions (planned / unplanned), and school absence. The introduction (to my reading) sets up for a study that will test associations between hospital admissions (independent variable) and school absences (dependent variable), but Methods suggests a range of potentially exploratory analyses. A couple of additional points here. I wonder whether classifying neurodisability as early or later (therefore creating three groups including the general population) adds a more complexity that is warranted. Secondly, there are, of course, known associations of socioeconomic disadvantage and frequency of hospital admissions. I’d suggest that the authors consider adjusting for these as socioeconomic variables as covariates in their proposed analysis. Of course, there are more disruptions to education because of health care other than hospital admissions. Primary care, disability related allied health interventions, and outpatient appointments are likely more numerous and potentially impacting - however, I think there is value in quantifying the impact of hospital admissions as an area that can potentially be avoided / minimised with good care (this is noted in the limitations).

 Some explanation of how the temporal relationships between hospital admissions and school absences are going to be considered would also be helpful. If a hospital admission happens on a weekend or during a school holiday. Or are hospital admissions going to used as a proxy for ill health?

 Abstract

 - A clear statement of the research question would be helpful here.

 Introduction

 - Also would benefit for clear statement of research question.

 Methods

 - Generally clear and well written.

 - Clarifying research question would help perhaps clarifying exposures (neurodisability, admissions?), covariates (sex, measures of socioeconomic disadvantage) and outcomes (school absences?).

 - As above, I’m not clear that splitting groups based on timing of neurodisability first admission is particularly meaningful (but happy to be educated).

 - Further thought about how to categorise the main outcome variable would be useful (is this going to be linear regression, poisson or logistic?)

 I look forward to hearing more about your project as it progresses, and will be interested in the results.

Is the study design appropriate for the research question?

Yes

Is the rationale for, and objectives of, the study clearly described?

Partly

Are sufficient details of the methods provided to allow replication by others?

Yes

Are the datasets clearly presented in a useable and accessible format?

Not applicable

Reviewer Expertise:

Paediatrics, disability, data linkage, health services research

I confirm that I have read this submission and believe that I have an appropriate level of expertise to confirm that it is of an acceptable scientific standard, however I have significant reservations, as outlined above.

# Reviewer report: Reviewer response for version 1

**DOI:** 10.3310/nihropenres.14717.r31663

**Published:** 2024-08-08

**Authors:** Rachel Knight Lozano

**Affiliations:** 1 University of Plymouth, Plymouth, England, UK

This paper describes the protocol for an England-based observational data linkage study of health and educational outcomes in primary school children with neurodisability.  

 The rationale for this study is clear, well-reasoned and compelling, acknowledging key issues of time spent in hospital and participation in school for children with neurodisability. The background highlights an important gap in record data linkage literature, in which healthcare usage and school attendance in children with neurodisability are each considered in isolation. Authors seek to address this through an observational study of health and educational outcomes. Although a clear aim of the wider research programme is stated, clear objectives of this specific observational study would add value to this protocol and support readers to align these with the proposed method and data analysis.

 The method is well written, structured through a clear framework of population, exposure and outcomes. Authors consider a range of relevant and inclusive health and educational records for data collection whilst acknowledging the anticipated limitations of data coverage beyond NHS-funded health and state-funded education. Alongside the outcomes of interest, authors consider important socioeconomic and demographic variables both in their data collection and analysis plan that may impact on their outcomes of interest in children with neurodisability.

 The timeframe for data collection appears well-reasoned, with consideration of the COVID pandemic and its impact on outcomes of health and education. It is not clear to the reader why the population age addresses primary school only; the health database records provide data sets from 1995 (ECHILD), 1997 (HES) and 1998 (mortality data), which presents an opportunity to extend the upper age limit age beyond 10/11, capturing important secondary school data as well. It would be useful to further understand the author's reasoning for this.

 The authors are very transparent about the risk of bias and limitations of this study. However, this observational study has potential to provide a platform for future research in the field of both health and educational neurodisability research, which is somewhat under-emphasised. More detail regarding anticipated opportunities for future research would add value to this protocol.

Is the study design appropriate for the research question?

Yes

Is the rationale for, and objectives of, the study clearly described?

Partly

Are sufficient details of the methods provided to allow replication by others?

Yes

Are the datasets clearly presented in a useable and accessible format?

Not applicable

Reviewer Expertise:

childhood neurodisability; chest health outcomes; prevention of chest-related illness; chest-related health usage

I confirm that I have read this submission and believe that I have an appropriate level of expertise to confirm that it is of an acceptable scientific standard.

# Reviewer report: Reviewer response for version 1

**DOI:** 10.3310/nihropenres.14717.r32443

**Published:** 2024-08-08

**Authors:** Rachael Marpole

**Affiliations:** 1 Perth Children's Hospital Foundation, Perth, Western Australia, Australia

Article on school absences in children with neurodisability from hospital admissions. 

 Introduction

 - Third paragraph: Change in school absences over time? (Early primary versus upper primary) - Is this being assessed as well?

 - Fourth paragraph: Will you look at neurodisability (diagnosed at any time) versus no neurodisability? Many of these conditions are present from birth, i.e. Autism, ADHD, and learning disability, it doesn't matter when their diagnosis is first recorded. Internalised autism is diagnosed later and not necessarily because it is less severe. 

 Methods - Study population

 - Why just singletons? Is it because it will be difficult to link hospitals with school data as they have the same birthdate and address? Multiples are more likely to be premature and have cerebral palsy and other complications. 

 Methods - Follow up

 - Will the dates of admissions be looked at? As most admissions happen in winter and more likely to be unplanned. 

 Methods - Exposure - neurodisability

 - This will miss children with neurodisability who are never admitted. Could you cross-reference with the school data to add children who are receiving special educational needs support?

 Methods - Outcomes -planned and unplanned hospital admissions 

 - Will admission days on weekends and school holidays be counted? As school can't be missed if it is not on. 

Is the study design appropriate for the research question?

Yes

Is the rationale for, and objectives of, the study clearly described?

Yes

Are sufficient details of the methods provided to allow replication by others?

Yes

Are the datasets clearly presented in a useable and accessible format?

Not applicable

Reviewer Expertise:

Cerebral palsy, Respiratory disease in neurodisability, paediatrics

I confirm that I have read this submission and believe that I have an appropriate level of expertise to confirm that it is of an acceptable scientific standard, however I have significant reservations, as outlined above.

# Reviewer report: Reviewer response for version 1

**DOI:** 10.3310/nihropenres.14717.r31671

**Published:** 2024-05-30

**Authors:** Laura Cowley

**Affiliations:** 1 Swansea University, Swansea, Wales, UK

This is a well-written and well-thought out study protocol that describes in detail a planned data linkage study to examine hospital admissions and school absences amongst children with neurodisability in England. I have a few suggestions which may improve the clarity of the manuscript. 

 Abstract

 In my opinion the background section of the abstract lacks detail on why the research questions are important to address (see also my comments on the Introduction, below). Similarly, the conclusions section of the abstract could state how the findings might contribute to policy and practice in the health and education fields for children with neurodisability. The word "group" should be "groups". 

 Introduction

 In general the introduction gives a good and succinct overview of the previous literature and the problem to be addressed, however I think what is missing is why this work is important and how the findings will help children with neurodisability to get better healthcare and education? It seems obvious and a bit of a foregone conclusion that children with neurodisability will have both a greater number of hospital admissions and a greater number of school absences compared to their non-disabled peers. The authors touch on why it is important to understand how health needs affect schooling, but there is a lack of detail about why it is important to compare hospital admissions for the two groups, and why it may be important to differentiate between planned and unplanned hospital admissions. How will knowing about these potential differences help to inform policy and practice and healthcare for children with neurodisability? 

 Last sentence - "provision of" should read "provision on"

 Methods

 My first thought is that one of the outcomes is hospital admissions, but neurodisability will be identified using ICD-10 codes, so by definition, children will have to have been admitted to hospital in order to be recorded as having a neurodisability. Is there circular reasoning here and can the authors comment on this? Is neurodisability more likely to be recorded in GP data than hospital data? Is it possible to also look at whether neurodisability is recorded in the education data, and to compare the diagnoses across the two datasets?

 The datasets, linkage and study population are all well-described and I like Table 1 to describe the cohort. Although there is justification for the end date of the cohort (2008) I might add in a justification for the start date (2003) too, for completeness. I am a little confused by the following sentence: "Children’s hospitalisation rates can be characterised across their primary school years whether they are in state-funded school (and so feature in a school census in NPD) or not." Surely this is not relevant, since children are only included in the cohort if their NHS record links to NPD, and NPD only includes state-funded schools anyway? The authors mention that they cannot see emigration in the data, but is it possible to see transition to home-schooling, independent non-state schooling or off-rolling in the data? Or is this just assumed by non-enrollment in a given year? 

 The sentence beginning "HES and NPD records" is very long, consider splitting it.

 The authors mention that "the codes used to identify cases were collated from published papers", please reference each of the papers that you sourced codes from. It is great to see that the code lists will be made available. 

 It would be good to see a bit more detail on the statistical analysis techniques that will be used. The authors mention regression, but what type of regression will be used? Logistic, Cox, Poisson? 

 Strengths, etc.

 I'm not sure I understand the following sentence: "Linkage of health and education data means that children with neurodisability can be identified by their hospital records." Neurodisability will only be defined using hospital records, you don't need to link to education data to do this? 

 "This study will also has limitations" - "has" should be "have".

Is the study design appropriate for the research question?

Yes

Is the rationale for, and objectives of, the study clearly described?

Partly

Are sufficient details of the methods provided to allow replication by others?

Partly

Are the datasets clearly presented in a useable and accessible format?

Not applicable

Reviewer Expertise:

Children's health and social care, data linkage, administrative data

I confirm that I have read this submission and believe that I have an appropriate level of expertise to confirm that it is of an acceptable scientific standard, however I have significant reservations, as outlined above.
